# Cases of Gastralgia and Irritable Stomach; Use of the Purified Oxide of Manganese

**Published:** 1864-07

**Authors:** 


### Selections.

### CASES OF GASTRALGIA AND IRRITABLE STOMACH; USE OF THF PURIFIED OXIDE OF MANGANESE.

From the perusal of Dr. Leared's observations on manganese as a remedy in disease of the stomach, Dr. Rogers was led to make a trial of this drug in cases of obstinate dyspepsia. Two of the patients in the following cases suffered from gastralgia; one from uterine derangement and sympathetic irritation of the stomach; and in the fourth, the discomfort after meals was probably caused by exalted sensibility of the mucous membrane in the manner described by Dr. Leared.

M. S., aged twenty-nine, a florid-looking married woman, had been under treatment for severe pain at the scrobiculus cordis, with occasional vomiting, since the latter part of November, 1863. Various remedies had been tried, none of them affording more than temporary relief. Dr. Rogers prescribed for the patient oxide of manganese in doses of ten grains three times a day.

Jan. 12th.—There was some difficulty in persuading her to persevere with the medicine, as she complained of its extreme "grittiness."

15th.—Has had no vomiting; less pain. To leave off eating supper, but before bedtime to take a dose of the mixture.

19th.—No pain after meals yesterday; bowels costive. To take a senna draught immediately.

29th.—Has not felt so "light and cheerful" for months, and is full of profuse expressions of gratitude. No nausea after taking the medicine.

Feb. 9th.—Discharged cured, but recommended to continue the mixture once a day for another week.

J. C., aged thirty-four, employed in the main drainage works. Had symptoms very similar to the above, for which he had been treated early last autumn at the hospital, but soon discontinued his attendance. He came again on Jan. 19th, and attributed all his "queer pain" to the beer he drank, but could not make up his mind to forego his favorite luxury. The manganese was given in doses of ten and fifteen grains three times a day, the diet not being altered in the least. On the 9th of February he was discharged well, having taken altogether sixty-two doses of the manganese.

H. D., aged twenty, a young woman of nervous temperament. Suffers from leucorrhœa, and a few months since was in the hospital under the care of Mr. Bird, who removed a vascular excrescence from the urethra. In addition to leucorrhœa, she complains of hypogastric pains, disrelish for food, and considerable thirst. There is a constant pain at the epigastrium, and occasionally rejection of the food immediately after it is swallowed. Dr. Rogers contemplated giving the oxide of silver, but the manganese has already done so much to relieve the gastric irritability that it is hoped a persistence in its use will effect a cure.

The fourth case is that of A. G., aged twenty-four, a laundress, who had constant heartburn and great discomfit after every meal, most likely attributable, as before stated, to unduly exalted sensibility of the mucous lining of the stomach, arising either from an irritable condition of the nervous filaments, or from excessive secretion of gastric juice, accompanied by increased vascularity. This patient was also troubled with shifting rheumatic pains. The uterine functions were regular; the bowels rather costive; tongue dirty. Bismuth gave no relief, and the mineral acids and bitter tonics were tried, but with no good effect. At times she was compelled to leave off work for a week together. Hydrocyanic acid and soda palliated the symptoms, but she was soon as bad as ever. On the 15th of January she commenced taking the oxide of manganese in ten-grain doses three times a day, and steadily continued it up to the 9th of February, when she stated that she was free from all uneasiness, and was about to take a new situation.

These four cases which are not picked ones, but such as come before the hospital physician every time he attends in the out-patient rooms, are intended further to illustrate the effects of a valuable agent which Dr. Leared has already so successfully employed in the treatment of forms of certain dyspepsia. Its comparative cheapness is, as Dr. Leared says, a great recommendation for hospital practice. Independently of this, however, there is a strong probability that Dr. Leared is correct in affirming its superiority to bismuth in many cases of gastralgia.

At the West London Hospital the purified oxide of manganese is procured as originally recommended from Messrs. Garden and Robbins, of Oxford street. It has not yet been exhibited in the form of powder, but as a draught; the compound tragacanth powder being used to suspend the manganese. It may be as well to mention that Dr. Neligan has recommended the *sulphate* of manganese in dyspectic affections and bilious disorders, not with a view of sheathing the mucous surface denuded of its epithelium, which is Dr. Leared's aim, but of promoting increased biliary secretion.—*London Lancet.*

